# Identification of candidate gene *FAM183A* and novel pathogenic variants in known genes: High genetic heterogeneity for autosomal recessive intellectual disability

**DOI:** 10.1371/journal.pone.0208324

**Published:** 2018-11-30

**Authors:** Megan McSherry, Katherine E. Masih, Nursel H. Elcioglu, Pelin Celik, Ozge Balci, Filiz Basak Cengiz, Daniella Nunez, Claire J. Sineni, Serhat Seyhan, Defne Kocaoglu, Shengru Guo, Duygu Duman, Guney Bademci, Mustafa Tekin

**Affiliations:** 1 John P. Hussmann Institute for Human Genomics, Miller School of Medicine, University of Miami, Miami, FL, United States of America; 2 Department of Pediatric Genetics, Marmara University Medical School, Istanbul, Turkey; 3 Eastern Mediterranean University Medical School, Cyprus, Mersin 10, Turkey; 4 Division of Developmental Pediatrics, Department of Pediatrics, Ankara University School of Medicine, Ankara, Turkey; 5 Division of Genetics, Department of Pediatrics, Ankara University School of Medicine, Ankara, Turkey; 6 Dr. John T. Macdonald Foundation Department of Human Genetics, Miller School of Medicine, University of Miami, Miami, FL, United States of America; Central South University Third Xiangya Hospital, CHINA

## Abstract

The etiology of intellectual disability (ID) is heterogeneous including a variety of genetic and environmental causes. Historically, most research has not focused on autosomal recessive ID (ARID), which is a significant cause of ID, particularly in areas where parental consanguinity is common. Identification of genetic causes allows for precision diagnosis and improved genetic counseling. We performed whole exome sequencing to 21 Turkish families, seven multiplex and 14 simplex, with nonsyndromic ID. Based on the presence of multiple affected siblings born to unaffected parents and/or shared ancestry, we consider all families as ARID. We revealed the underlying causative variants in seven families in *MCPH1* (c.427dupA, p.T143Nfs*5), *WDR62* (c.3406C>T, p.R1136*), *ASPM* (c.5219_5225delGAGGATA, p.R1740Tfs*7), *RARS* (c.1588A>G, p.T530A), *CC2D1A* (c.811delG, p.A271Pfs*30), *TUSC3* (c.793C>T, p.Q265*) and *ZNF335* (c.808C>T, p.R270C and c.3715C>A, p.Q1239K) previously linked with ARID. Besides ARID genes, in one family, affected male siblings were hemizygous for *PQBP1* (c.459_462delAGAG, p.R153Sfs*41) and in one family the proband was female and heterozygous for X-chromosomal *SLC9A6* (c.1631+1G>A) variant. Each of these variants, except for those in *MCPH1* and *PQBP1*, have not been previously published. Additionally in one family, two affected children were homozygous for the c.377G>A (p.W126*) variant in the *FAM183A*, a gene not previously associated with ARID. No causative variants were found in the remaining 11 families. A wide variety of variants explain half of families with ARID. *FAM183A* is a promising novel candidate gene for ARID.

## Introduction

Intellectual disability (ID) is an early-onset neurodevelopmental disorder affecting 1% of the general population [1]. ID is characterized by a significant impairment in cognitive ability and adaptive behavior affecting memory, language, problem solving, and visual comprehension, which can lead to impairments in activities of daily living such as self-care and interpersonal communication.

Environmental factors, such as teratogens, infections, malnutrition, and neurological trauma as well as genetic conditions can cause ID. The literature supports a strong genetic etiology for ID, with a varying proportion of cases (ranging from 17% to 50%) being attributable to genetic causes [2–5]. Variants in more than 1,000 genes have been connected to ID [6]. Support for rare *de novo* variants as a major cause of ID in simplex cases have been reported [7]. Microarray and exome sequencing have demonstrated the importance of *de novo* copy number variations (CNVs) and single‐nucleotide variations (SNVs) in ID. While research elucidating chromosomal aberrations, CNVs, autosomal dominant, and X-linked variants as causes for ID has been well established, it was not until recently that studies focusing on autosomal recessive forms of ID (ARID) have gained attention [6,8]. ARID either presents as the sole clinical feature (nonsyndromic) or with additional features (syndromic), and it is extremely heterogeneous [9,10]. To date, there are fifty-five genes in the phenotypic series [PS249500] for non-syndromic ARID in the Online Mendelian Inheritance in Man (OMIM) database [6]. ARID appears to be a common form of ID, an unsolved healthcare problem creating an enormous socioeconomic burden on society, especially in the underdeveloped countries where there is a high rate of consanguinity [8,11]. To reveal the causative variants in ARID, we performed Whole Exome Sequencing (WES) in 21 families affected by non-syndromic ID; these families either had multiple affected family members or pedigrees suggestive of consanguinity.

## Methods

### Ethics statement

This study was approved by the University of Miami Institutional Review (USA), the Marmara University Medical School Ethics Committee (Turkey), and the Ankara University Medical School Ethics Committee (Turkey). A signed informed consent form was obtained from each participant or, in the case of a minor, from the parents.

### Subjects

We included families with ID and a pedigree structure suggestive of autosomal recessive inheritance. These were a mix of multiplex families with parental consanguinity (7 families), simplex families with reported parental consanguinity (5 families), or parental origin from a small town (9 families). All families were from Turkey and evaluated at Marmara University Medical School or Ankara University Medical School. All affected children received a thorough physical examination and were evaluated by a pediatrician, geneticist, and neurologist, when available.

Patients with major anomalies, syndrome specific phenotypic features, and specific neurological signs were excluded from the study. Patients with non-specific minor anomalies (e.g., clinodactyly) and neurological signs (e.g., seizures) were not excluded from the classification of non-syndromic ID, as most patients with ID have such findings. DNA was extracted from patient blood samples for genomic analysis.

### Whole exome sequencing and bioinformatics analysis

In 6 families, we performed WES in two affected siblings, and in 15 families, only the probands were sequenced. Agilent SureSelect Human All Exon 50 Mb was used for the capture and HiSeq 2000 was used for the sequencing per our previously published protocol [12]. We filtered variants based on minor allele frequency of <0.005 for recessive and <0.0005 for dominant using ExAC (http://exac.broadinstitute.org/; accessed 08/15/2018) and GnomAD (http://gnomad.broadinstitute.org/; accessed 08/15/2018), a genotype quality (GQ) score >35 for the variant quality, and minimum read depth of ≥ 8. Combined Annotation Dependent Depletion (CADD: http://cadd.gs.washington.edu/info), Sorting Intolerant from Tolerant (SIFT: http://sift.jcvi.org/) and Mutationtaster (http://www.mutationtaster.org/) were used for the *in silico* prediction. ClinVar (https://www.ncbi.nlm.nih.gov/clinvar/) and Human Gene Mutation Database (HGMD: http://www.hgmd.cf.ac.uk/ac/index.php) were used for the mutation databases. Genomic evolutionary rate profiling (GERP: http://mendel.stanford.edu/SidowLab/downloads/gerp/index.html) score was used to determine variant conservation. While autosomal recessive inheritance with both homozygous and compound heterozygous models were chosen during the initial analysis, all inheritance modes were subsequently investigated. Missense, nonsense, splice site, in-frame INDEL and frame-shift INDELs were selected. We searched for variants in genes already implicated in ID. These genes were retrieved from OMIM with query words including “intellectual disability” or “mental retardation” or “microcephaly” or “cognitive impairment” in Clinical Synopsis (S1 Table). We used CoNIFER (Copy Number Inference From Exome Reads) [13] and XHMM (eXome-Hidden Markov Model) [14] to detect Copy Number Variants (CNVs) [15]. For the CNV analysis, we evaluated genes in our list. We filtered out common CNVs using our WES controls that consists of >500 individuals and Database of Genomic Variants (DGV; http://dgv.tcag.ca/dgv/app/home). ACMG 2015 Guidelines were used for the variant interpretation [16]. Sanger sequencing was used for variant confirmation and co-segregation (dx.doi.org/10.17504/protocols.io.tzxep7n) (S2 Table).

For those families without causative variants in known ID genes, we obtained runs of homozygosity (>2 MB) shared by affected members of each family (S3 Table). Only homozygous INDELs, single nucleotide variants, and CNVs mapping to runs of homozygosity were included in the analysis.

## Results

On average, 99%, 94% and 89% of the captured regions were covered by 1X, 5X, and 10X reads, respectively. Average read depth was 59X in our cohort.

### Known variants associated with intellectual disability

We identified two variants that have previously been reported to cause ID in two genes (Table 1). In family S223, one male proband (II:1) was homozygous for c.427dupA (p.T143Nfs*5) in *MCPH1*. In family S25, both affected brothers (II:1 and II:2) were found to be hemizygous for c.459_462delAGAG (p.R153Sfs*41) in *PQBP1*.

**Table 1:** Summary of the identified variants in this study.

Family ID	Gene	MIM#	Associated Syndrome	MIM#	Zygosity	NM#	cDNA	Protein	Reference	ExAC	gnomAD	CADD	GERP RS	Mutation Taster	SIFT	ClinVar	ACMG
AU10	*CC2D1A*	610055	Mental retardation, autosomal recessive 3	608443	HM	NM_017721.4	c.811delG	p.A271Pfs*30	This study	N/A	N/A	33	5.3699	N/A	N/A	N/A	P
MR25	*FAM183A*	N/A	Novel candidate gene	N/A	HM	NM_001101376.2	c.377G>A	p.W126*	This study	0.0008695	0.0009992	38	4.07	DC	N/A	N/A	N/A
MR32	*TUSC3*	601385	Mental retardation, autosomal recessive 7	611093	HM	NM_006765.3	c.793C>T	p.Q265*	This study	N/A	N/A	46	4.2399	DC	N/A	N/A	P
S25	*PQBP1*	300463	Mental retardation, X-linked, Renpenning type	309500	HZ	NM_001032383.1	c.459_462delAGAG	p.R153Sfs*41	Kalscheuer et al 2003 [17]	N/A	N/A	35	N/A	N/A	N/A	P	P
S223	*MCPH1*	607117	Microcephaly 1, primary, autosomal recessive	251200	HM	NM_024596.4	c.427dupA	p.T143Nfs*5	Trimborn et al.	N/A	N/A	28.8	4.21	N/A	N/A	P/LP	P
									2004 [18]								
S228	*WDR62*	613583	Microcephaly 2, primary, autosomal recessive, with or without cortical malformations	604317	HM	NM_001083961.1	c.3406C>T	p.R1136*	This study	0.000008267	0.00001806	35	0.266	DC	N/A	N/A	P
S234	*SLC9A6*	300231	Mental retardation, X-linked syndromic, Christianson type	300243	HT	NM_006359.2	c.1631+1G>A	Splice	This study	N/A	N/A	28.4	5.0399	DC	N/A	P	LP
S236	*ZNF335*	610827	Microcephaly 10, primary, autosomal recessive	615095	HT	NM_022095.3	c.808C>T	p.R270C	This study	0.0006979	0.0005315	24.1	3.8199	DC	DM	VUS	VUS
					HT	NM_022095.3	c.3715C>A	p.Q1239K	This study	0.00004253	0.00001629	23.8	5.03	DC	DM	N/A	VUS
S243	*ASPM*	605481	Microcephaly 5, primary, autosomal recessive	608716	HM	NM_018136.4	c.5219_5225delGAGGATA	p.R1740Tfs*7	This study	N/A	N/A	35	N/A	N/A	N/A	N/A	P
S244	*RARS*	107820	Leukodystrophy, hypomyelinating, 9	616140	HM	NM_002887.3	c.1588A>G	p.T530A	This study	N/A	N/A	23.6	4.7899	DC	DM	N/A	VUS

**HM:** Homozygous, **HZ:** Hemizygous, **HT:** Heterozygous, **N/A:** Not Available, **DC:** Disease Causing, **DM:** Damaging, **P:** Pathogenic, **LP:** Likely Pathogenic, **VUS:** Variant of Uncertain Significance, **ACMG:** American College of Medical Genetics Guidelines

### Novel variants in known genes associated with intellectual disability

**Fig 1:**
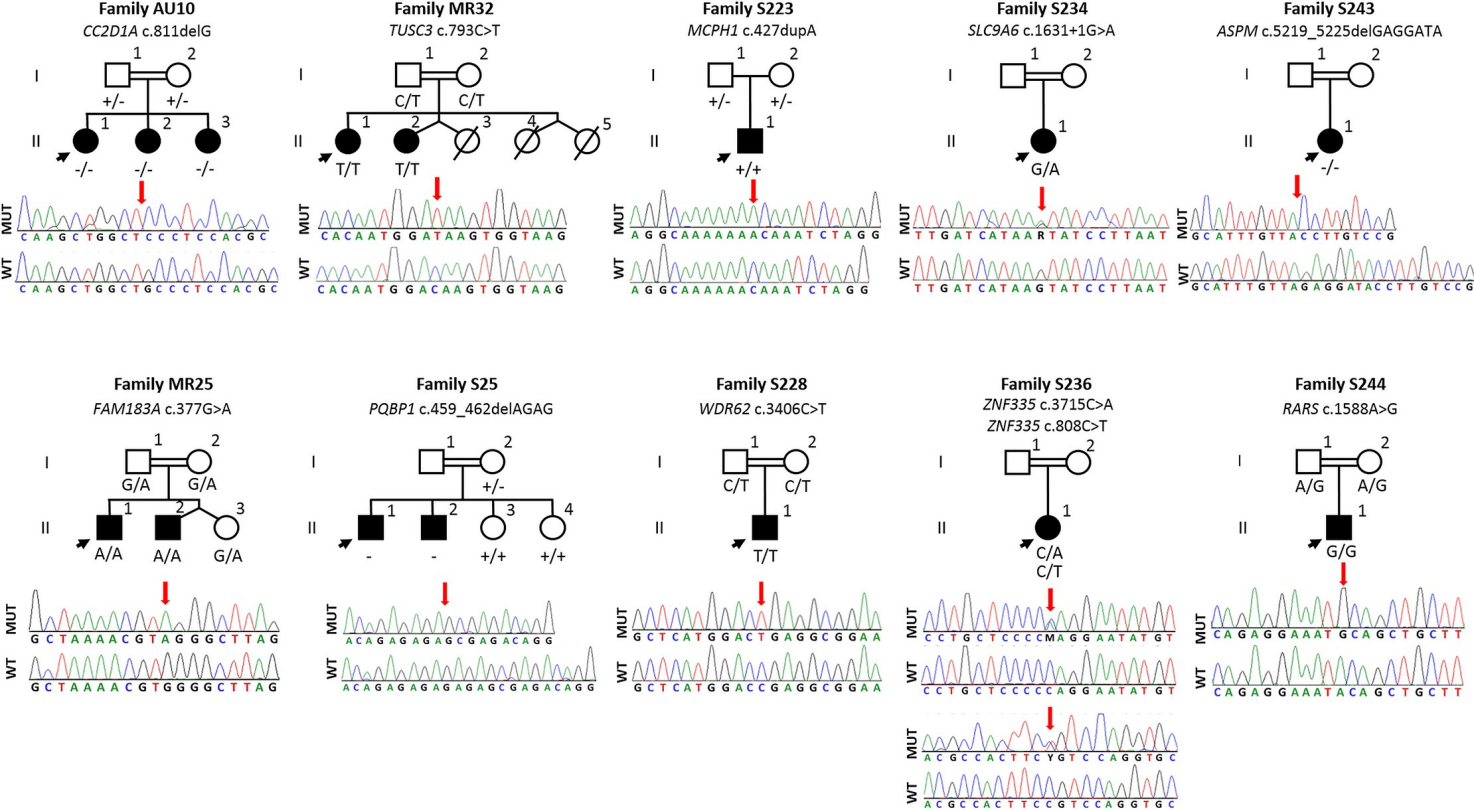
The electropherograms of the identified variants and pedigrees of the families in this study. WT: Wildtype, MUT: Mutant.

Whole exome sequencing of the probands resulted in identification of seven novel variants in seven genes known to cause ID (Table 1 and S4 Table). These variants were associated with varying phenotypes (Table 2). Variant segregation was confirmed with Sanger sequencing (Fig 1). Homozygous variants in *CC2D1A* and *ASPM* led to frameshift mutations. Additionally, homozygous nonsense variants in *TUSC3* and *WDR62* and a heterozygous variant in *SLC9A6* occurring at a splice site were identified. Lastly, a homozygous substitution in *RARS* and two heterozygous variants in *ZNF335* were found. These results are described in more detail in Table 1.

**Table 2:** Phenotypic features of probands with causative variants.

Family-individual ID	GENE	Simplex/multiplex	Parental consanguinity reported	Sex	Age at last exam	Growth	Craniofacial	Cardiovascular	Abdomen/ GU	Skeletal	Skin/hair/ nails	Neurologic
AU10-II:1	*CC2D1A*	Mx	Y	F	11y	Normal height and head circumference	No distinctive findings	N	N	N	N	ID, autistic spectrum disorder, seizures
AU10-II:2				F	*8 y*	Normal height and head circumference	No distinctive findings	N	N	N	N	ID, history of global developmental delay with autistic features
AU10-II:3				F	*5 y*	Normal height and head circumference	No distinctive findings	N	N	N	N	Developmental delay especially speech delay with autistic features, seizures
MR25-II:1	*FAM183A*	Mx	Y	M	12 y	Normal height	Borderline microcephaly, upslanting palpebral fissures, strabismus, retinal pigmentation	N	N	N	N	IQ 48 Autism Spectrum Disorder
MR25-II:2			Y	M	14 m	Normal height	Upslanting palpebral fissures, L ptosis, blue sclera, ears slightly large	N	Recurrent urinary tract infections	N	N	Mild developmental delay
MR32-II:1	*TUSC3*	Mx	Y	F	8.5 y	Short stature	Microcephaly, strabismus	N	N	N	N	ID, agitations
MR32-II:2			Y	F	3.5 y	Borderline short stature	Microcephaly, strabismus	N	N	N	N	Global developmental delay
S25-II:1	*PQBP1*	Mx	Y	M	20 y	Short stature, lean body build	Microcephaly, brachycephaly, long and narrow face, short philtrum, cupped ears, broad nasal bridge	Pectus excavatum	N	N	N	Moderate ID, history of global delay especially in speech
S25-II:2				M	10 y	Short stature	Microcephaly, long face, broad nasal bridge	N	N	N	N	Moderate ID
S223-II:1	*MCPH1*	Sx	N	M	1.5 m	Normal height	Microcephaly, narrow frontal area, long philtrum, big ears	N	N	Simian crease	Seborrheic dermatitis	Cortical atrophy, deep sulci on cranial MRI
S228-II:1	*WDR62*	Sx	Y	M	1y	Normal height	Microcephaly, bilateral epicanthus	N	N	N	N	Mild global developmental delay
S234-II:1	*SLC9A6*	Sx	Y	F	3y	Normal height	Microcephaly, square jaw, ophthalmoplegia, L ear anomaly, skin tag	Narrow chest				global developmental delay
S236-II:1	*ZNF335*	Sx	Y	F	2 m	Normal height	Severe microcephaly, short neck, big ears, high palate, deep philtrum, micrognathia	Peripheral pulmonary stenosis	Bilateral mild pelviectasis	Long 2^nd^ toe	Bilateral single palmar crease	seizures, cranial MRI: corpus callosum agenesis, hydrocephaly, colpocephaly
S243-II:1	*ASPM*	Sx	Y	F	2 y	Normal height	Severe microcephaly, narrow frontal area, big ears	N	N	N	N	Cranial CT: cortical atrophy, deep sulci
S244-II:1	*RARS*	Sx	Y	M	8 m	Normal height	Severe microcephaly, big ears, micrognathia		Bilateral inguinal hernia	N	Sandal gap	Developmental delay, no eye tracking of light or object

**Blank:** limited clinical information, **Sx:** simplex, **Mx:** multiplex, **y:** years, **m:** months, **M:** male, **F:** female, **N:** no abnormal clinical findings

### Mutated *FAM183A* as a causative candidate for intellectual disability

Whole exome sequencing revealed a nonsense variant (c.377G>A; p.W126*) in *FAM183A*, a novel candidate gene for ARID. Two siblings, MR25-II:1 and MR25-II:2, were homozygous for the variant, and in the family, the variant segregated in an autosomal recessive fashion (Fig 1).

## Discussion

We have identified causative variants in 10 out of 21 Turkish families with nonsyndromic ARID.

11 families remained unsolved in our cohort (S1 Fig and S5 Table).

### Known variants associated with intellectual disability

The two families with previously reported causative variants for ID are phenotypically consistent with the previously described findings. Both brothers (II:1 and II:2) in family S25 show findings indicative of Renpenning syndrome [MIM 309500], an X-linked condition [17]. Zhang et al (2017) discusses the molecular pathogenesis of mutations in *PQBP1*, pointing toward the promotion of ubiquitin-mediated degradation of fragile X mental retardation protein (FMRP) resulting in synaptic dysfunction [19]. WES, elucidating the presence of a known causative variant, accompanied with phenotypic data confirmed a diagnosis of Microcephaly 1 in proband S223-II:1 [18].

### Novel variants in known genes associated with intellectual disability

*CC2D1A* variants are associated with ARID (MRT3) [MIM 608443]. This disorder is characterized by significantly below average general intellectual functioning associated with impairments in adaptive behavior which manifest during the developmental period. Analysis in Drosophila links *CC2D1A*, a member of the mammal lethal giant discs (lgd) protein family, to endosomal trafficking of Notch proteins, well known transmembrane receptors that regulate cell fate during development [20]. Basel-Vanagaite et al. (2006) reported homozygosity for a protein truncating mutation in *CC2D1A* in affected members of 9 consanguineous Israeli-Arab families with nonsyndromic ID [21]. The homozygous deletion at c.811delG (p.A271Pfs*30) of *CC2D1A* found in family AU10 is the fifth variant associated with ID reported in this gene.

*TUSC3* variants are known to cause Mental Retardation, Autosomal Recessive 7 (MRT7) [MIM 611093]. Including the finding reported here, four independent autosomal recessive variants in *TUSC3* are known to cause ARID [22–24]. The exact molecular pathogenesis is unknown. One study suggests the involvement of N-glycosylation in higher brain functions [24], while another postulates disturbed magnesium levels due to *TUSC3* impairment may play a role in the pathogenesis of intellectual disability [22]. This novel nonsense variant at c.793C>T (p.Q265*) in *TUSC3* in family MR32 further demonstrates the significance of this gene’s association with ARID.

Seventeen variants in numerous domains of *WDR62* have been reported to be associated with Microcephaly 2, primary, autosomal recessive, with or without cortical malformations (MCPH2) [MIM 604317] [25–28]. Cellular studies indicate that *WDR62* is a crucial protein in enabling spindle poles to position the cytokinetic furrow and prolong neural precursor generation, a process that is uniquely vital to the proper growth of the human cerebral cortex [25]. The attributes of the proband, S228-II:1, described in Table 2, are consistent with this phenotype. This novel homozygous nonsense variant in *WDR62*, c.3406C>T (p.R1136*) along with the phenotype of the proband further implicate this gene’s involvement in ARID.

Variants in *SLC9A6* are associated with Mental retardation, X-linked syndromic (MRXSCH), Christianson type [MIM 300243]. *SLC9A6* encodes a monovalent sodium-selective sodium/hydrogen exchanger (NHE) found in the membranes of intracellular organelles such as mitochondria and endosomes; NHEs participate in a wide array of essential cellular processes [29]. MRXSCH is characterized by profound ID, epilepsy, ataxia, and microcephaly. The phenotype of the female proband (S234-II:1), described in Table 2, is consistent with the presentation of MRXSCH. Linkage analysis and DNA sequencing of families with MRXSCH have identified multiple variants in the *SLC9A6*, the majority of which are truncating and/or splice mutations [30–34]. The novel heterozygous splice-site variant, c.1631+1G>A in *SLC9A6*, adds to the body of literature on pathogenic splice-site variants reported in this gene.

*ZNF335* mutations are associated with Microcephaly 10, primary, autosomal recessive (MCPH10) [MIM 615095]. One homozygous variant in *ZNF335* (S236-II:1), causing both a missense change and a splice site defect, is linked to this syndrome [35]. This same study demonstrated that *ZNF335* deficiency causes disrupted neuronal proliferation and differentiation *in vitro* and *in vivo* mouse models [35]. Both discovered variants in our study are predicted to be disease causing and are likely to result in a hypomorphic variant of *ZNF335*. This, in combination with the gene’s association with MCPH10 and altered neuronal growth *in vitro* and *in vivo*, make it likely that these mutations are the cause of this family’s ID.

Variants in *ASPM* are associated with Microcephaly 5, primary, autosomal recessive (MCPH5) [MIM 608716]. ASPM has been implicated in the determination of human cerebral cortical size via maintenance of a cleavage plane orientation allowing for symmetric, proliferative division of neuroepithelial cells during brain development [36]. Several aberrations in *ASPM* have been reported in the literature with a clear majority resulting in premature termination [37–41]. Similarly to previously reported variants, the novel homozygous deletion c.5219_5225delGAGGATA (p.R1740Tfs*7) found in *ASPM* (S243-II:1) leads to a premature stop codon, suggesting that this variant is involved in ARID.

Lastly, variants in *RARS* are associated with Leukodystrophy, hypomyelinating, 9 (HLD9) [MIM 616140]. *RARS* encodes the cytoplasmic arginyl‐tRNA synthetase, an enzyme essential for RNA translation and a key player in myelination, among the subunits of the multisynthase complex [42]. Thus, mutations in *RARS* cause a hypomylenating disorder of the central nervous system. Previously identified variants in *RARS* causing the HLD9 phenotype are compound heterozygous mutations [42]. In contrast to previously identified variants in *RARS*, the novel variant found in family S244 is a homozygous missense mutation. After exome sequencing and segregation analysis, these mutations are the only variants that segregate with the family’s phenotype. Therefore, we propose that this homozygous missense variant has a role in ARID and is the most likely cause of ID this family.

### Mutated *FAM183A* as a causative candidate for intellectual disability

In family, MR 25, there were two siblings with ID, low set ears, and microcephaly along with other correlating phenotypic features (Table 2, Fig 1). The two affected children are homozygous for a *FAM183A* variant (c.377G>A; p.W126*). This variant, found in MR25-II:1 and MR25-II:2, is located in the longest shared homozygous genomic region in these siblings (S6 Table). *FAM183A* is expressed in human brain, including the hippocampus, caudate nucleus, and medulla oblongata [43]. Five heterozygous large deletions including *FAM183A* are reported in the DECIPHER database, (https://decipher.sanger.ac.uk/; accessed 07/08/2018). Three out of five variants are found in patients with intellectual disability, developmental delay, or microcephaly; however, the variants’ pathogenicities are not known [44].

The pathogenicity models, expression in brain tissue, and validated autosomal recessive segregation of this variant with the phenotype in family MR 25 support a role for this homozygous nonsense variant as contributing to the inheritance of ID. More research, including functional studies, should be done to further support the role of *FAM183A* in the pathogenesis of ID.

In 10 out of 21 families investigated (47.6%) we identified likely disease-causing DNA variants. Although CNVs are important cause of ID, we did not detect any CNV in our cohort. As expected for affected offspring of healthy consanguineous parents, the clear majority of these were autosomal recessive defects. This yield is comparable to other recent investigations in highly consanguineous populations [7,45–50].

## Supporting information

S1 Table List of the genes used for the analysis in our cohort. The gene list is created by using the command *((((mental retardation[Clinical Synopsis]) OR microcephaly[Clinical Synopsis]) OR intellectual disability[Clinical Synopsis]) OR cognitive impairment[Clinical Synopsis]) AND “prefix pound”[Properties]* within the web site https://www.ncbi.nlm.nih.gov/omim (accessed 10/02/2018). After curation of the gene list, we analyzed single nucleotide variants, INDELs and copy number variants in the gene list. Due to limitation of the next generation sequencing method that we used, we did not analyze the repeat expansions.
(XLSX)

S2 Table Sequences of the primers used for the Sanger sequencing. (XLSX)

S3 Table Parameters used for detecting homozygous runs by using Enlis software (https://www.enlis.com/). (XLSX)

S4 Table Chromosomal position of the identified variants (hg19). (XLSX)

S5 Table Phenotypic features of the unsolved probands. (XLSX)

S6 Table Autozygous regions shared by MR25- II:1 and II:2 (hg19). (XLSX)

S1 Fig Pedigrees of the unsolved families. (TIF)
