# Supplementary figures and images for: Identification of candidate gene FAM183A and novel pathogenic variants in known genes: High genetic heterogeneity for autosomal recessive intellectual disability
Source: PLoS One. 2018 Nov 30;13(11):e0208324. doi: 10.1371/journal.pone.0208324 (PMC6267965; doi:10.1371/journal.pone.0208324)

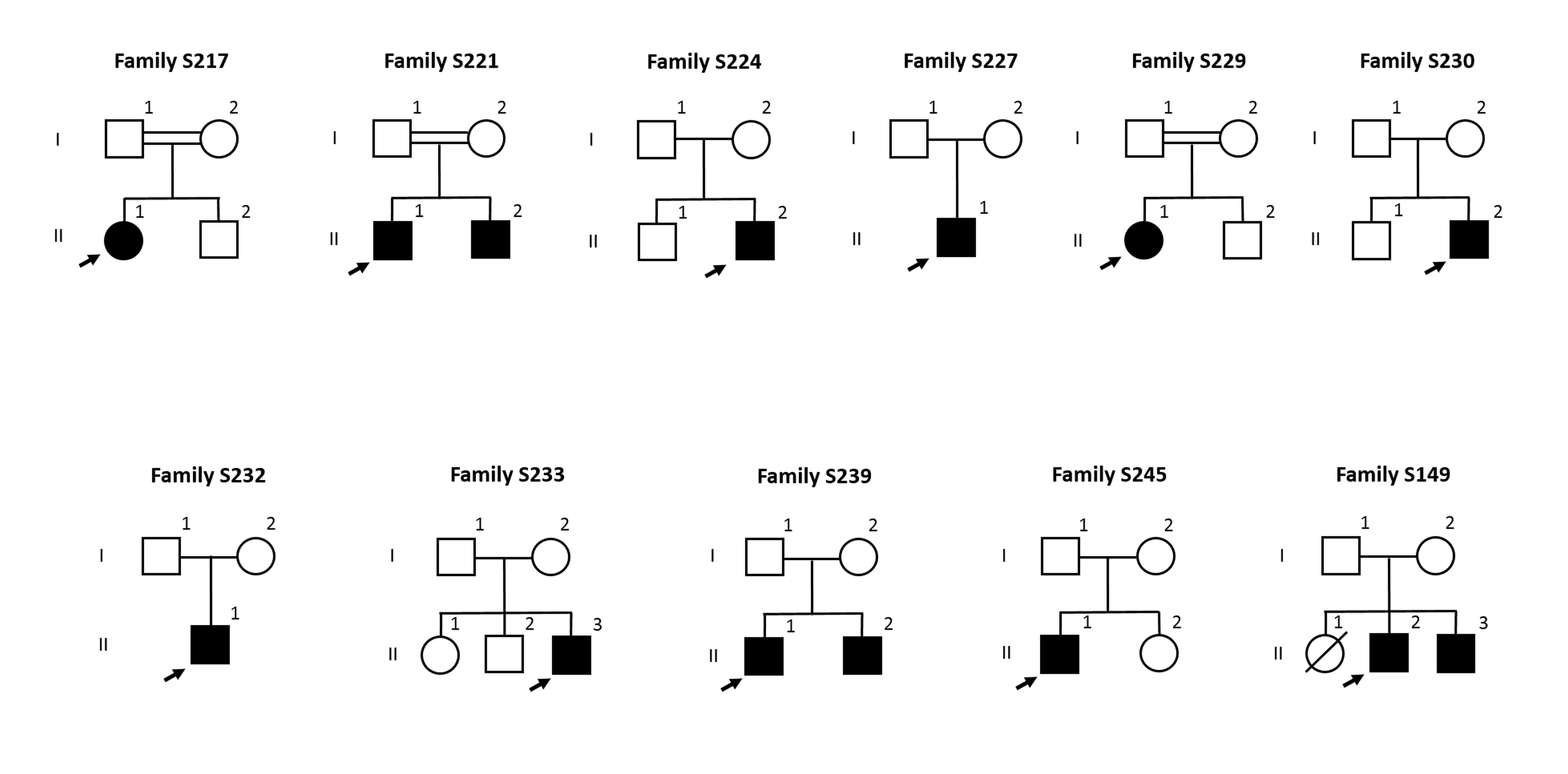

Supplement: S1 Fig — (TIF) [file pone.0208324.s007.tif]
